# Racial Disparities in Analgesic and Psychiatric Medication Use During End-Of-Life Care in Advanced-Stage Colorectal Cancer: A Retrospective Cohort Study

**DOI:** 10.1158/2767-9764.CRC-25-0164

**Published:** 2025-07-08

**Authors:** John M. Allen, Olga M. Trejos Kweyete, Yi Guo, Jiang Bian, Xiwei Lou, Sherise C. Rogers, Lisa Scarton, David L. DeRemer, Diana J. Wilkie

**Affiliations:** 1 Department of Pharmacy Practice, Purdue University College of Pharmacy, West Lafayette, Indiana.; 2 Department of Pharmacotherapy and Translational Research, University of Florida College of Pharmacy, Orlando, Florida.; 3 Department of Health Outcomes & Biomedical Informatics, University of Florida College of Medicine, Gainesville, Florida.; 4 Department of Biostatistics and Health Data Science, Indiana University School of Medicine, Indianapolis, Indiana.; 5 Division of Hematology & Oncology, University of Florida College of Medicine, Gainesville, Florida.; 6 Department of Family, Community, and Health Systems Science, University of Florida College of Nursing, Gainesville, Florida.; 7 Department of Pharmacotherapy and Translational Research, University of Florida College of Pharmacy, Gainesville, Florida.; 8 Department of Biobehavioral Nursing Science, University of Florida College of Nursing, Gainesville, Florida.

## Abstract

This study examined racial and ethnic disparities in the use of analgesic and psychiatric medications during end-of-life care among Medicare beneficiaries with advanced-stage colorectal cancer. Using the Surveillance, Epidemiology, and End Results–Medicare–linked database from 2005 to 2017, we identified 28,212 patients with stage IV colorectal cancer who died within 1 year of diagnosis. Multivariable logistic regression models were used to assess differences in medication use by race and ethnicity. Compared with non-Hispanic White patients, Black patients had significantly lower odds of opioid use [adjusted OR (aOR) = 0.86; 95% confidence interval (CI), 0.80–0.93] and overall analgesic use, whereas Hispanic patients had higher use of opioids (aOR = 1.12; 95% CI, 1.03–1.22) and non-opioid analgesics (aOR = 1.22; 95% CI, 1.06–1.40). Asian patients had increased non-opioid use (aOR = 1.71; 95% CI, 1.44–2.03) and decreased skeletal muscle relaxant use (aOR = 0.59; 95% CI, 0.43–0.82). Across all minority groups, psychiatric medication use was consistently lower than in non-Hispanic White patients. These disparities persisted after adjusting for demographic, clinical, and socioeconomic factors. Findings highlight the urgent need for equitable, culturally responsive symptom management strategies to improve the quality of end-of-life care in this population.

**Significance:**

We identified significant disparities in the use of analgesic and psychiatric medications among patients with advanced-stage colorectal cancer. Our findings are significant given the emerging importance of symptom management on health-related quality of life and survival. Future research is needed to understand causal factors, their influence on patient-reported outcomes such as symptom relief, and the development of strategies to close these medication use gaps.

## Introduction

Colorectal cancer is a leading cause of cancer-related death in the United States, with substantial pain and psychologic burden, particularly during end-of-life (EoL) care (1). Racial and ethnic disparities in colorectal cancer outcomes are well documented, yet little is known about disparities in the use of supportive care medications—specifically analgesics and psychiatric medications—during this critical phase of care. This study aims to describe patterns of analgesic and psychiatric medication use by race and ethnicity among Medicare beneficiaries with advanced-stage colorectal cancer during the final year of life.

Pain is a common symptom in colorectal cancer, affecting more than 70% of patients, particularly in advanced stages (2). Chronic and persistent pain often accompanies disease progression and treatment-related side effects, contributing to both physical and emotional distress. Unrelieved pain can exacerbate psychologic symptoms such as anxiety, depression, agitation, and delirium. In addition, colorectal cancer itself is associated with a high prevalence of psychologic distress, with 13% to 25% of patients experiencing depression or anxiety (3). Pharmacologic management of these symptoms includes analgesics, antidepressants, anxiolytics, and antipsychotics.

Effective symptom management is essential for optimizing quality of life and improving health outcomes. Studies have linked better symptom control to improved survival in patients with advanced cancer (4–6). Health-related quality of life (HRQoL) is also a strong prognostic indicator, with higher HRQoL associated with better treatment response (7). Conversely, inadequate management of pain and other symptoms contributes to treatment interruptions (8) and worse clinical outcomes (9), underscoring the need for high-quality symptom management and equitable access to analgesic and psychiatric medications.

Racial disparities in HRQoL among patients with cancer, especially during EoL care, have been documented in multiple studies (10). These disparities are driven by complex factors, including social determinants of health, differences in care quality, and sociocultural influences (11–13). For example, Black patients with cancer are more likely to require emergency department visits and undergo intensive treatments in the last 6 months of life compared with White patients, suggesting lower HRQoL at EoL (14). Clinical guidelines on palliative care highlight the urgent need for further research to address these disparities (15). Prior studies have reported racial disparities in the use of analgesic and psychiatric medications in various cancers, including pancreatic, breast, and brain metastases (16–18). Analgesic and psychiatric medications are critical components of symptom management during EoL care, making equitable access to these therapies an essential element of high-quality care and efforts to reduce cancer health disparities.

However, little is known about the extent of analgesic and psychiatric medication use during EoL care in patients with advanced-stage colorectal cancer. This study aims to fill that gap by describing these patterns by race and ethnicity in Medicare beneficiaries during the last year of life.

## Materials and Methods

### Study design

This retrospective cohort study utilized the Surveillance, Epidemiology, and End Results (SEER)–Medicare–linked database from 2005 to 2017. The SEER–Medicare database is a population-based collection of SEER cancer registry data that include clinical and demographic information on cancer cases (RRID: SCR_006902) linked to Medicare claims for healthcare services. Prescription medication data were obtained through Medicare Part D claims. The datasets used for this study are available upon approval of a research protocol from the NCI; detailed instructions for data access are available at https://healthcaredelivery.cancer.gov/seermedicare/obtain/. This study was reviewed and approved as exempt by the University of Florida Institutional Review Board (study #IRB201701945). The article was prepared in accordance with the Strengthening the Reporting of Observational Studies in Epidemiology guidelines (19).

### Study population

Participants were identified using International Classification of Diseases 9 and International Classification of Diseases 10 codes specific to colorectal cancer. Race and ethnicity were classified based on medical records and administrative data and included non-Hispanic White, non-Hispanic Black/African American, non-Hispanic Asian/Pacific Islander, non-Hispanic American Indian/Alaska Native, and Hispanic (of any race). Eligible patients had continuous Medicare Part A and Part B coverage from colorectal cancer diagnosis until death. Exclusion criteria included death more than 1 year after colorectal cancer diagnosis (16, 18), lack of Medicare Part D coverage in the 3 months prior to death, missing race and ethnicity information, diagnosis at stage 0 to IIIC, or age younger than 65 years at diagnosis. Data coarsening and suppression were applied to comply with the NCI’s SEER–Medicare data cell size suppression policy (*n* < 11; ref. 20).

### Pain and psychiatric medications

Given the substantial pain and psychiatric symptom burden experienced by patients with advanced-stage colorectal cancer during EoL, the study focused on supportive care medications, defined as analgesics and psychiatric medications used to manage these symptoms. The EoL period was defined as the 12 months preceding death (16, 18). Medication use was defined as having at least one Medicare claim for a medication of interest during the EoL period. Only outpatient prescription claims were included in the analysis. Analgesic medications were categorized into four classes: opioid analgesics, non-opioid analgesics, skeletal muscle relaxants, and neuropathic analgesics. Psychiatric medications included antidepressants, anxiolytics, and antipsychotic agents (16, 18). A full list of medications is provided in Supplementary Table S1.

### Statistical methodology

Categorical variables were compared across racial and ethnic groups using *χ*^2^ tests, whereas continuous variables were compared using ANOVA and the Kruskal–Wallis test. Logistic regression models were developed for each medication class to assess the association between medication use and race/ethnicity. The primary multivariable model (model 1) was adjusted for age, sex, marital status, ZIP code–level high school completion rate, ZIP code–level median household income, patient residence, year of colorectal cancer diagnosis, Charlson comorbidity index at diagnosis, and Medicare hospice care claims. A secondary model (model 2) excluded socioeconomic status variables (education and income) in alignment with the National Academies of Medicine’s definition of healthcare disparities (21, 22). Non-Hispanic White patients were the reference group for all comparisons. Results are presented as covariate-adjusted ORs (aOR) with 95% confidence intervals (CI). A two-sided *P* value < 0.05 was considered statistically significant. Based on a conservative estimate of 10% medication use (23), a sample size of 13,380 patients was required to detect a 15% difference in medication use. Analyses were conducted using SAS version 9.4.

### Data availability

The datasets used and analyzed in this study are available upon request from the SEER–Medicare–linked database (https://healthcaredelivery.cancer.gov/seermedicare/); detailed instructions for data access are available at https://healthcaredelivery.cancer.gov/seermedicare/obtain/.

## Results

### Patient demographics

Of the 474,531 patients with colorectal cancer identified, 446,319 were excluded based on the study’s predefined criteria, leaving 28,212 patients in the final analytic cohort. The primary reasons for exclusion were lack of continuous Medicare coverage within 3 months of diagnosis and a stage 0 to IIIC colorectal cancer diagnosis. Supplementary Fig. S1 provides details on study inclusion and exclusion criteria.

Among the included patients, 54.8% were women and 38.6% were aged 80 years or older. The racial and ethnic distribution was as follows: non-Hispanic White, 72.8%; non-Hispanic Black/African American, 12.7%; Hispanic (any race), 9.5%; non-Hispanic Asian/Pacific Islander, 4.7%; and non-Hispanic American Indian/Alaska Native, 0.35%. Because of small sample size, non-Hispanic American Indian/Alaska Native patients were excluded from the multivariable logistic regression analysis. Table 1 presents clinical and demographic characteristics of the study population by race and ethnicity.

**Table 1:** Clinical and demographic characteristics of patients with advanced-stage CRC stratified by race and ethnicity

Characteristic	White (*n* = 20,541)	Black/African American (*n* = 3,575)	Hispanic (*n* = 2,682)	Asian/Pacific Islander (*n* = 1,312)	American Indian/Alaska Native (*n* = 101)	All patients (*n* = 28,212)
Sex, % (*n*)						
Female	54.8 (11,265)	58.9 (2,104)	49.9 (1,339)	52.3 (686)	55.5 (56)	54.8 (15,450)
Age group, % (*n*)^a^						
65–74	39.7 (8,134)	48.7 (1737)	44.5 (1,190)	39.2 (514)	54.5 (55)	41.3 (11,630)
75–80	20.3 (4,152)	19.7 (702)	20.3 (542)	18.2 (238)	24.8 (25)	20.1 (5,659)
>80	40 (8,199)	31.7 (1,130)	35.3 (943)	42.6 (558)	20.8 (21)	38.6 (10,851)
Marital status, %, (*n*)						
Single/divorced	35.9 (7,371)	47.4 (1,696)	37.6 (1,009)	35.8 (470)	55.5 (56)	37.6 (10,602)
Married/partnered	32.3 (6,642)	20.6 (736)	33.5 (900)	44.5 (584)	26.7 (27)	31.5 (8,889)
Unknown	31.8 (6,528)	32 (1,143)	28.9 (774)	19.7 (258)	17.8 (18)	30.9 (8,721)
Percent with high school as the highest grade level completed, median % (IQR)^b^	27.9 (20.8–34.5)	29.7 (24.7–34.9)	24.9 (21–28.7)	22.2 (17.5–26)	28.8 (23.9–33.3)	27.3 (21.1–33.9)
Household income (per 1K USD), median (IQR)^b^	57.8 (44.8–76.6)	41.7 (31.9–55.6)	50 (38.6–64.6)	60.5 (46.9–80.6)	45.9 (39.3–59.9)	55.1 (42.3–73.8)
Residence, % (*n*)						
Metro	85 (17,465)	91.3 (3,266)	96.1 (2,579)	98.4 (1,291)	78 (78)	87.5 (24,679)
Urban–suburban	13.2 (2,714)	7.7 (274)	c	c	c	11.1 (3,130)
Rural	1.8 (361)	1.0 (35)	c	c	c	1.4 (401)
Year of CRC diagnosis, median (IQR)	2012 (2009–2014)	2012 (2009–2014)	2012 (2009–2014)	2012 (2009–2014)	2013 (2010–2015)	2012 (2009–2014)
Charlson comorbidity index, %, (*n*)						
0–2	80.7 (16,585)	75.5 (2,701)	81.3 (2,182)	80.3 (1,053)	79.2 (80)	80.1 (22,601)
>2	19.3 (3,956)	24.5 (874)	18.7 (501)	19.7 (259)	20.8 (21)	19.9 (5,611)
Medicare hospice claim, % (*n*)	69.2 (14,212)	61.2 (2,186)	65.6 (1,759)	54.4 (714)	64.3 (65)	67.1 (18,936)

Abbreviation: CRC, colorectal cancer.

a Age at pancreatic cancer diagnosis

b Zip code level

c Data suppressed to prevent identification of cell count values of 11 or less.

### Analgesic and psychiatric medication use by class

Overall, racial and ethnic differences were observed across multiple medication classes, with notable patterns in both analgesic and psychiatric medication use. In model 1, opioid use varied across racial and ethnic groups. Black patients had lower utilization compared with non-Hispanic White patients (aOR = 0.86; 95% CI, 0.80–0.93), whereas Hispanic patients had higher utilization (aOR = 1.12; 95% CI, 1.03–1.22). The use of non-opioid analgesics was higher among Hispanic (aOR = 1.22; 95% CI, 1.06–1.40) and Asian (aOR = 1.71; 95% CI, 1.44–2.03) patients, whereas Black patients had similar utilization compared with non-Hispanic White patients. Skeletal muscle relaxant use was lower among Asian patients (aOR = 0.59; 95% CI, 0.43–0.82), with no significant differences observed in Black or Hispanic patients. No racial or ethnic differences were observed in neuropathic analgesic use relative to non-Hispanic White patients. Overall, Black patients were less likely to receive any analgesic medication compared with non-Hispanic White patients (aOR = 0.87; 95% CI, 0.80–0.94), whereas Hispanic patients were more likely to receive any analgesic (aOR = 1.15; 95% CI, 1.05–1.26). No significant differences in overall analgesic use were observed between Asian and non-Hispanic White patients (aOR = 1.12; 95% CI, 0.99–1.27). Model 2 produced results comparable with model 1, with the exception of a significant increase in neuropathic analgesic use among Black patients relative to non-Hispanic White patients. No other meaningful differences were noted between models.

With regard to psychiatric medications, all minority groups generally had lower use compared with non-Hispanic White patients across drug classes. For antidepressants, lower use was observed in Black (aOR = 0.54; 95% CI, 0.50–0.60), Hispanic (aOR = 0.82; 95% CI, 0.75–0.90), and Asian (aOR = 0.48; 95% CI, 0.42–0.56) patients. Anxiolytic use was also lower among Black (aOR = 0.43; 95% CI, 0.38–0.49), Hispanic (aOR = 0.76; 95% CI, 0.67–0.85), and Asian (aOR = 0.45; 95% CI, 0.37–0.55) patients. For antipsychotic use, only Asian patients had lower utilization compared with non-Hispanic White patients (aOR = 0.76; 95% CI, 0.60–0.98); no differences were observed in Black or Hispanic patients. When examining overall psychiatric medication use, all racial and ethnic minority groups had lower utilization compared with non-Hispanic White patients (Black: aOR = 0.52; 95% CI, 0.48–0.57; Hispanic: aOR = 0.79; 95% CI, 0.73–0.86; and Asian: aOR = 0.49; 95% CI, 0.43–0.56). Model 2 results were consistent with model 1 for antidepressant and anxiolytic use. The previously observed lower antipsychotic use in Asian patients was no longer statistically significant in model 2 (aOR = 0.80; 95% CI, 0.62–1.02). No differences or trends were identified based on the year of colorectal cancer diagnosis. Table 2 provides detailed unadjusted and adjusted medication utilization patterns across racial and ethnic groups.

**Table 2:** Use of analgesic and psychiatric medication by race and ethnicity

Drug class	Unadjusted model			Adjustment model 1			Adjustment model 2		
	Black/African American	Hispanic	Asian/Pacific Islander	Black/African American	Hispanic	Asian/Pacific Islander	Black/African American	Hispanic	Asian/Pacific Islander
Analgesic medications									
Opioids	1.10 (1.02–1.20)	0.91 (0.85–0.98)	0.90 (0.80–1.00)	**0.86** (0.80–0.93)	**1.12** (1.03–1.22)	1.01 (0.90–1.14)	**0.87** (0.80–0.93)	**1.11** (1.02–1.21)	0.97 (0.86–1.09)
Non-opioid analgesics	1.14 (1.01–1.29)	1.21 (1.06–1.38)	1.69 (1.43–1.99)	1.06 (0.94–1.21)	**1.22** (1.06–1.40)	**1.71** (1.44–2.03)	1.12 (0.99–1.28)	**1.25** (1.09–1.43)	**1.70** (1.44–2.01)
Skeletal muscle relaxants	1.08 (0.93–1.27)	0.99 (0.83–1.20)	0.58 (0.42–0.79)	0.93 (0.79–1.10)	0.97 (0.81–1.18)	**0.59** (0.43–0.82)	1.01 (0.86–1.18)	0.99 (0.82–1.19)	**0.59** (0.43–0.82)
Neuropathic analgesics	1.19 (1.07–1.32)	1.12 (0.99–1.26)	1.04 (0.88–1.24)	1.05 (0.94–1.18)	1.09 (0.95–1.24)	** **1.08 (0.90–1.29)	**1.12** (1.00–1.25)	1.12 (0.99–1.27)	1.07 (0.90–1.28)
Receipt of any analgesic medication	0.94 (0.88–1.02)	1.17 (1.08–1.21)	0.99 (0.89–1.11)	**0.87** (0.80–0.94)	**1.18** (1.08–1.29)	1.12 (0.99–1.26)	**0.90** (0.83–0.97)	**1.18** (1.08–1.28)	1.07 (0.95–1.21)
Psychiatric medications									
Antidepressants	0.58 (0.53–0.63)	0.80 (0.73–0.88)	0.47 (0.40–0.54)	**0.54** (0.50–0.60)	**0.82** (0.75–0.90)	**0.48** (0.42–0.56)	**0.54** (0.50–0.59)	**0.81** (0.74–0.89)	**0.48** (0.42–0.56)
Anxiolytics	0.47 (0.42–0.53)	0.78 (0.69–0.88)	0.49 (0.40–0.59)	**0.43** (0.38–0.49)	**0.76** (0.67–0.85)	**0.45** (0.37–0.55)	**0.41** (0.37–0.47)	**0.74** (0.66–0.84)	**0.46** (0.38–0.56)
Antipsychotics	1.11 (0.97–1.27)	1.00 (0.86–1.18)	0.79 (0.60–0.97)	0.96 (0.83–1.11)	0.94 (0.80–1.11)	**0.76** (0.60–0.98)	1.03 (0.89–1.18)	0.99 (0.85–1.17)	0.80 (0.62–1.02)
Receipt of any psychiatric medication	0.57 (0.53–0.62)	0.79 (0.73–0.86)	0.49 (0.43–0.55)	**0.52** (0.48–0.57)	**0.79** (0.73–0.86)	**0.49** (0.43–0.56)	**0.53** (0.49–0.57)	**0.79** (0.72–0.86)	**0.49** (0.43–0.56)

Data are presented as OR (95% CI). All comparisons were made to non-Hispanic White patients. In model 1, results were adjusted for age, sex, marital status, ZIP code–level high school completion, ZIP code–level median household income, patient residence, year of colorectal cancer diagnosis, Charlson comorbidity index, and Medicare hospice claim. In model 2, ZIP code–level high school completion and ZIP code–level median household income were excluded. Bold text in adjusted model results indicates a *P* value < 0.05. Blue-shaded cells indicate lower medication use, and red-shaded cells indicate higher medication use.

## Discussion

This study identified significant racial and ethnic disparities in the use of analgesic and psychiatric medications during EoL care among Medicare beneficiaries with advanced-stage colorectal cancer. Black patients were less likely to receive opioids and overall analgesics, whereas Hispanic patients had higher use of both opioids and non-opioid analgesics. Asian patients demonstrated higher use of non-opioid analgesics but lower use of skeletal muscle relaxants, with no difference in opioid use compared with non-Hispanic White patients. Psychiatric medication use was consistently lower across all minority groups. These findings highlight the complex and nuanced patterns of analgesic and psychiatric medication use by race and ethnicity and underscore the need for culturally responsive interventions to promote equitable symptom management at EoL.

An interesting finding was the lack of differences in neuropathic analgesic use across racial and ethnic groups despite prior studies suggesting that Black patients may be at higher risk for chemotherapy-induced neuropathy (24). This may reflect similar exposure to chemotherapy regimens across groups, as suggested by prior research (25). However, known risk factors for neuropathy—such as higher body mass index and diabetes—are more common among racial minorities (25, 26), which raises concerns about potential undertreatment of neuropathy-related pain. Further research is needed to explore these patterns and their impact on symptom control and quality of life.

Colorectal cancer–related pain is multifactorial, involving nociceptive, neuropathic, and psychologic components. It can arise from tumor biology (e.g., kallikrein–kinin system activation and inflammatory cytokines) or treatment-related factors (e.g., chemotherapy, surgery, and radiation; refs. 23, 27–30). Managing colorectal cancer–related pain requires a nuanced approach that integrates these factors, with opioids being the mainstay of pharmacologic therapy and neuropathic agents used to treat chemotherapy-induced neuropathy. Although effective pain management improves HRQoL and physical functioning (31, 32), evidence linking opioid use to survival benefits remains inconsistent (33). Guidelines emphasize opioids as the standard treatment for cancer pain using a tiered management strategy (34, 35). Notably, we also observed a bidirectional pattern in analgesic use. Although Hispanic and Asian patients had higher use of non-opioid analgesics, Black patients had lower use of opioids and analgesics overall. These findings challenge assumptions about universal undertreatment of pain among minority groups and suggest that disparities are not uniform across populations. Differences in prescribing practices, cultural preferences, symptom burden, or access to hospice services may contribute to these patterns. Further research is needed to understand the drivers of these disparities and their implications for patient outcomes, including symptom relief and HRQoL.

Our finding of uniformly lower psychiatric medication use across all minority groups is concerning. These patterns may reflect a combination of cultural beliefs, stigma, differences in perceived need, provider prescribing behavior, and barriers to access—all of which can influence treatment uptake (36). Prior studies have shown that untreated psychiatric conditions in patients with cancer are associated with higher mortality, whereas psychiatric treatment is linked to improved outcomes (37). These results highlight the critical need to address psychiatric care disparities as part of comprehensive EoL symptom management.

Compared with prior research, our study provides important new insights into symptom management disparities in advanced-stage colorectal cancer. Although previous studies have examined disparities in treatment access and outcomes across racial groups, few have focused specifically on disparities in analgesic and psychiatric medication use during EoL care for patients with colorectal cancer (38–40). This is a critical gap as colorectal cancer is associated with a high symptom burden compared with other cancers (41). Our findings build upon prior work showing disparities in supportive care medication use in other cancer populations, including pancreatic, breast, and brain metastases (16–18). Interestingly, we found that disparities in colorectal cancer were less pronounced than those observed in pancreatic cancer. One possible explanation is differences in hospice use: hospice use was lower among racial and ethnic minorities in the advanced-stage colorectal cancer population (61.5%) compared with our previous research in pancreatic cancer (65.5%; ref. 18). Understanding the direct impact of this difference on pain and psychiatric medication use warrants further investigation.

The differences in medication use between patients with advanced-stage colorectal cancer and pancreatic cancer were observed despite both being gastrointestinal cancers and having similar 5-year relative survival rates (1, 42, 43). This underscores the nuances in medication use across cancer types. Outside of gastrointestinal cancers, prior studies have also identified racial disparities in supportive care medication use in advanced-stage breast cancer, particularly in antidepressants and sleep aids, with Black patients having lower use than White patients (17). Similar disparities have been observed in patients with brain metastases, for whom minority groups were less likely to receive psychiatric medications and showed mixed patterns in analgesic use (16). In a broader study of opioid access among Medicare beneficiaries with various cancers, Black and Hispanic patients were less likely to receive opioids and had lower-intensity dosing compared with White patients (44). Our study provides a more nuanced understanding of pain and psychiatric medication use disparities in the context of advanced-stage colorectal cancer among Medicare beneficiaries.

Our study has several strengths, including the use of a large, nationally representative database and an analytic approach that intentionally excluded socioeconomic variables, consistent with the National Academies of Medicine’s definition of healthcare disparities (21, 22). This approach allowed us to more directly examine the independent effect of race and ethnicity on medication use.

However, our study is not without limitations. Our reliance on Medicare claims data limits generalizability to younger patients. Nevertheless, the study population represents most patients with colorectal cancer as incidence is highest in adults over 65 years of age (1). We did not capture medication use outside of Medicare, although approximately 94% of adults over 65 years of age have Medicare coverage (45), limiting the risk of missing data. We could not assess inpatient medication use, nonprescription therapies, or non-pharmacologic treatments such as acupuncture or counseling. Race and ethnicity classifications are based on administrative data, but prior research suggests high concordance with self-reported information (46). A key limitation is the focus on the EoL period and the inability to distinguish new psychiatric prescriptions from ongoing maintenance therapy. Given known differences in baseline psychiatric medication use across racial and ethnic groups, our findings may partly reflect preexisting disparities rather than new prescriptions initiated during EoL care. Finally, our study did not examine sociocultural or psychosocial factors that may influence medication use. Future research should explore these factors to inform tailored interventions.

In conclusion, we identified significant racial and ethnic disparities in the use of analgesic and psychiatric medications during EoL care in patients with advanced-stage colorectal cancer. These findings underscore the need for targeted research to understand the causes of these disparities, assess their impact on patient-reported outcomes, and develop strategies to ensure equitable access to symptom management for patients with advanced-stage colorectal cancer.

## Supplementary Material

Supplemental Figure S1 Supplemental Figure S1. Study Inclusion Diagram

Supplemental Table S1 Supplemental Table S1. Supportive Care Medications by Drug Class

## Data Availability

The datasets used to conduct this study are available upon approval of a research protocol from the NCI. Instructions for obtaining these data are available at https://healthcaredelivery.cancer.gov/seermedicare/obtain/. The collection of cancer incidence data used in this study was supported by the California Department of Public Health pursuant to California Health and Safety Code Section 103885; Centers for Disease Control and Prevention’s National Program of Cancer Registries, under cooperative agreement 1NU58DP007156; and the NCI’s SEER Program under contract HHSN261201800032I awarded to the University of California, San Francisco, contract HHSN261201800015I awarded to the University of Southern California, and contract HHSN261201800009I awarded to the Public Health Institute. The ideas and opinions expressed herein are those of the authors and do not necessarily reflect the opinions of the State of California, Department of Public Health, the NCI, and the Centers for Disease Control and Prevention or their contractors and subcontractors.
