# Supplementary material for: Racial Disparities in Analgesic and Psychiatric Medication Use During End-Of-Life Care in Advanced-Stage Colorectal Cancer: A Retrospective Cohort Study
Source: Cancer Res Commun. 2025 Jul 8;5(7):1095–101. doi: 10.1158/2767-9764.CRC-25-0164 (PMC12234946; doi:10.1158/2767-9764.CRC-25-0164)
Supplement: Supplemental Figure S1 — Study Inclusion Diagram [file crc-25-0164_supplemental_figure_s1_suppsf1.docx]

***Supplemental Figure 1. Study Inclusion Diagram***

CRC Stage IV

(n= 28, 212)

Age ≥ 65 years old

(n=136, 014)

CRC patients with death within 12 months of diagnosis

(n=156, 897)

Continuous Medicare D coverage 3 months prior to diagnosis
(n = 255, 730)

Patients with CRC, 2005-2017

(n = 474, 531)

Patients excluded (No continuous Medicare D coverage 3 months prior to diagnosis
(n = 218, 801)

Patients excluded (Death outside of 12 month window)
(n = 98, 833)

Patients excluded

(unknown race/ethnicity)
(n = 4, 641)

Known race and ethnicity

(n= 152, 256)

Patients excluded (Age < 65)
(n = 16,242)

Patients excluded

(CRC Stage 0-IIIC at diagnosis)
(n = 107, 802)
