# Supplementary material for: Racial Disparities in Analgesic and Psychiatric Medication Use During End-Of-Life Care in Advanced-Stage Colorectal Cancer: A Retrospective Cohort Study
Source: Cancer Res Commun. 2025 Jul 8;5(7):1095–101. doi: 10.1158/2767-9764.CRC-25-0164 (PMC12234946; doi:10.1158/2767-9764.CRC-25-0164)
Supplement: Supplemental Table S1 — Supportive Care Medications by Drug Class [file crc-25-0164_supplemental_table_s1_suppst1.docx]

**Supplemental Table 1. Supportive Care Medications by Drug Class**

| **Non-opioid analgesics** | **Opioid analgesics** | **Skeletomuscular relaxants** | **Neuropathic analgesics** | **Antidepressants** | | **Anxiolytics** | **Antipsychotics** |
| --- | --- | --- | --- | --- | --- | --- | --- |
| Celecoxib  Diclofenac  Diflunisal  Etodolac Salsalate  Fenoprofen  Flurbiprofen  Ketoprofen  Ketorolac  Indomethacin  Meloxicam  Nabumetone  Piroxicam | Fentanyl  Codeine-acetaminophen  Hydrocodone-Acetaminophen  Hydrocodone  Hydromorphone  Methadone  Morphine  Meperidine  Oxycodone  Oxycodone-Acetaminophen  Oxycodone-Aspirin  Oxymorphone Butorphanol  Buprenorphine  Tapentadol  Tramadol  Nalbuphine | Baclofen  Carisoprodol  Cyclobenzaprine  Metaxalone  Methocarbamol  Orphenadrine  Tizanidine | Duloxetine  Gabapentin  Pregabalin | Milnacipran  Venlafaxine  Levomilnacipran  Desipramine  Nortriptyline  Amitriptyline  Amoxapine  Bupropion  Citalopram  Clomipramine  Desipramine  Desvenlafaxine  Doxepin  Fluoxetine  Fluvoxamine  Imipramine  Isocarboxazid | Escitalopram  Levomilnacipran  Maprotiline  Milnacipran  Mirtazapine  Nefazodone  Nortriptyline  Paroxetine  Phenelzine  Protriptyline  Selegiline  Sertraline  Tranylcypromine  Trazodone  Trimipramine  Venlafaxine  Vilazodone  Vortioxetine | Alprazolam  Clonazepam  Chlordiazepoxide  Clorazepate  Diazepam  Lithium  Lorazepam  Oxazepam  Phenelzine | Aripiprazole  Chlorpromazine  Haloperidol  Olanzapine  Trifluoperazine  Quetiapine  Risperidone  Olanzapine |
